# Education research - Understanding the factors involved in inpatient communication for orthopedic trainees

**DOI:** 10.1016/j.amsu.2021.103079

**Published:** 2021-11-19

**Authors:** Drew Daniel, Raffi Avedian, Tyler Johnson, John B. Michaud, Barbette Weimer-Elder, Merisa Kline, Aussama K. Nassar

**Affiliations:** a Stanford University, School of Medicine, CA, USA; b Department of Orthopedic Surgery, Stanford University, CA, USA; c Department of Internal Medicine, Stanford University, CA, USA; d Stanford Health Care, Patient Experience, Physician Partnership Team, Stanford, CA, USA; e Department of Surgery, Stanford University, CA, USA

**Keywords:** Orthopedic surgery, Resident education, Communication, Relationship-centered care, Needs assessment, ICNAS, Inpatient Communication Needs Assessment Survey, RCC, Relationship-Centered Care, PGY, Post-Graduate Year, ICS, Interpersonal and Communication Skills, ACGME, Accreditation Council for Graduate Medical Education, EMR, Electronic Medical Record

## Abstract

**Background:**

“Interpersonal and Communication Skills” (ICS) is a core competency set forth by the ACGME. No structured curriculum exists to train orthopedics residents in ICS.

**Methods:**

Twenty-four out of thirty-five orthopedics residents completed the survey (69%). The survey had the following domains: [1] Demographics, [2] Communication Needs/Goals, and [3] Communication Barriers.

**Results:**

Eighty-three percent of respondents wanted to improve their communication skills and their patient's experience. Interns-PGY4s wanted to improve on similar specific communication skills. All residents desired training in conflict management.

**Conclusion:**

There is a need among orthopedics residents for a communication skills curriculum early in residency training, specifically in conflict management.

## Highlights

• In procedural training programs (including Orthopedics), there is less focus on non-technical skills acquisition.
• When surveyed, 83% of orthopedic trainees perceived a need to improve their communication skills.
• All orthopedic trainees wanted training in conflict management.

## 1 Introduction

The ACGME requires that residency programs teach and evaluate residents in six core competencies, including Interpersonal and Communication Skills (ICS). Effective communication has been shown to boost patient and physician satisfaction [1,2], reduce physician burnout [3], and reduce malpractice lawsuits [4]. Currently, orthopedics residents are taught communication skills implicitly through direct observation of a preceptor and role modeling [5]. Such implicit learning, however, suffers from the uneven skills of preceptors and time constraints that limit such interactions. Our review of the literature did not find evidence of explicit, curriculum-based inpatient communication training for orthopedics residents other than a pilot program focused on communication specific towards geriatric patients [6].

We adapted Relationship-Centered Care (RCC) as the communication framework to use in the design of this needs assessment. RCC can be defined as care in which all participants appreciate the importance of their relationships with one another [7]. There are four foundational principles of RCC: (1) that relationships in healthcare ought to include the personhood of the participants, (2) that affect and emotion are important components of these relationships, (3) that all healthcare relationships occur in the context of reciprocal influence, and (4) that the formation and maintenance of genuine relationships in healthcare is morally valuable [7]. Implementation of RCC in pediatric inpatient units led to decreased preventable adverse events (e.g., delay in treatment causing patient harm) and increased family-reported understanding of information discussed during rounds [8]. For physicians, RCC leads to greater personal accomplishment, team cohesion, and workplace satisfaction [9], as well as a decrease in the chance of being sued [[10], [11], [12]].

Historically, orthopedic surgeons have been regarded as “high tech, low touch” physicians. Although orthopedic surgeons are given high ratings for their technical skills in the operating room, only 21% of patients reported satisfactory physician communication and only 37% reported their physicians as being caring and compassionate [13]. For comparison, 75% of orthopedic surgeons believed that they communicated effectively with their patients [13]. Potentially contributing to these perceptions, orthopedists show empathy infrequently in patient encounters and scored the second lowest out of all specialties on empathy [14,15]. Regarding residents, senior orthopedic residents scored lower on emotional intelligence than junior residents, indicating that residency training, as currently structured, may not be providing residents the socioemotional and communication training needed for RCC [16].

The purpose of this study is to explore the inpatient communication needs of orthopedics residents at our institution. This needs assessment will serve as the core foundation in developing a targeted inpatient communication curriculum, utilizing the principles of RCC, for orthopedics residents.

## 2 Methods

A needs assessment was performed to determine the perceived communication needs of orthopedics residents in the inpatient setting. This survey was adapted from a survey created for general surgery residents and modified with expert input from a panel of educational experts [17]. All thrity-five orthopedic surgery residents at our institution were invited to voluntarily complete an electronic Qualtrics survey. Residents were contacted up to four times over five weeks. Residents who completed the survey were compensated with a gift card funded by our institution's Department of Orthopaedic Surgery. The study was approved by our institution's University Institutional Review Board as an exempt protocol.

The Inpatient Communication Needs Assessment Survey (ICNAS) examined the following sections: [1] Demographics, [2] Communication Needs/Goals, and [3] Communication Barriers. Sections [2] and [3] of the survey utilized 5-point frequency and Likert scales respectively. For analysis of the frequency scale, we grouped the five subcategories into two main categories, with “almost always” and “usually” grouped together and “half the time”, “occasionally”, and “rarely” grouped together. Similarly, for analysis of the Likert scale, we grouped the five subcategories into two main categories with “strongly agree” and “agree” grouped together and “neutral”, “disagree”, and “strongly disagree” grouped together. The data were summarized with descriptive statistics and sub-analysis was done by PGY level, with a focus on the response differences between junior and senior residents.

## 3 Results

**Fig. 1:**
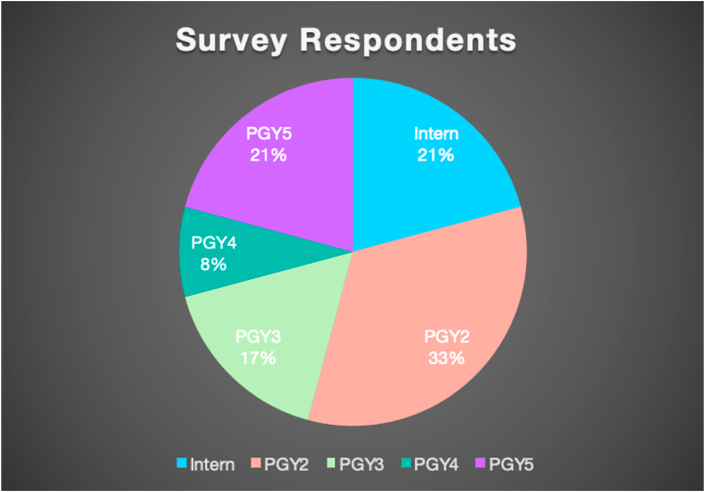
Survey respondents sorted by PGY (color). (For interpretation of the references to colour in this figure legend, the reader is referred to the Web version of this article.)

Twenty-four out of thirty-five orthopedic surgery residents completed the ICNAS (69% response rate). Survey respondents (Fig. 1) consisted of five interns (21%), eight PGY2s (33%), four PGY3s (17%), two PGY4s (8%), and five PGY5s (21%). The mean age (SD) of survey respondents was 29 (2.1) with 71% identifying as male and 29% identifying as female. Of all survey respondents, 58% identified as White, 9% identified as Black, 13% identified as Asian, and 21% identified as Other.

Survey results showed only one resident (4%) had participated in prior communication skills training, while 83% of residents wanted to both improve their own communication skills and needed to improve their patient's experience. Additionally, 75% of residents almost always/usually regard communication training as being relevant (Table 1). The majority of residents (79%) preferred an online method of course delivery. No residents currently practice RCC and 46% were not at all familiar with RCC.

**Table 1:** Communication Practices (all respondents).

	Almost always/usually (%)	Half the time/occasionally/rarely (%)
*I regard communication training as being relevant*	18 (75%)	6 (25%)
*I negotiate with patients to establish the agenda*	7 (29%)	17 (71%)
*I consistently elicit patients' concerns*	5 (21%)	19 (79%)
*I offer opportunities for patients to express emotions*	8 (33%)	16 (67%)
*I quickly establish rapport*	18 (75%)	6 (25%)
*I use open-ended questions to explore patients' perspectives*	10 (42%)	14 (58%)
*I encourage my patients to summarize what we discussed*	3 (12%)	21 (88%)
*I acknowledge patients' emotions with empathic responses*	11 (46%)	13 (54%)
*I share information in small “chunks”*	18 (75%)	6 (25%)
*I consistently check my patients' understanding*	10 (42%)	14 (58%)

**Fig. 2:**
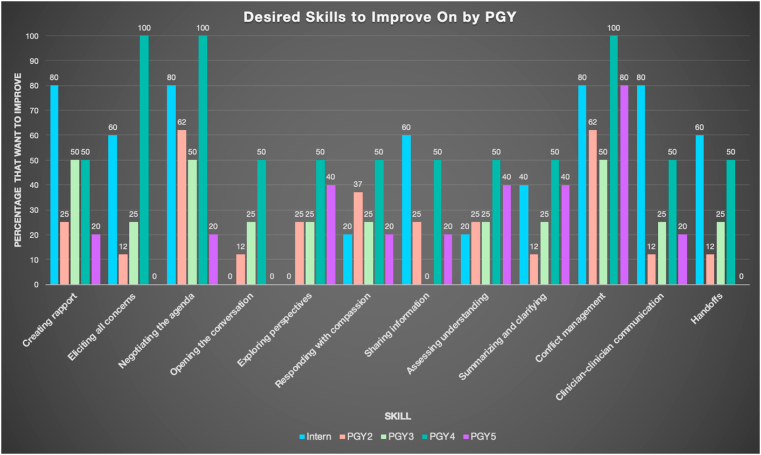
Desired skills to improve on by PGY (color). (For interpretation of the references to colour in this figure legend, the reader is referred to the Web version of this article.)

**Fig. 3:**
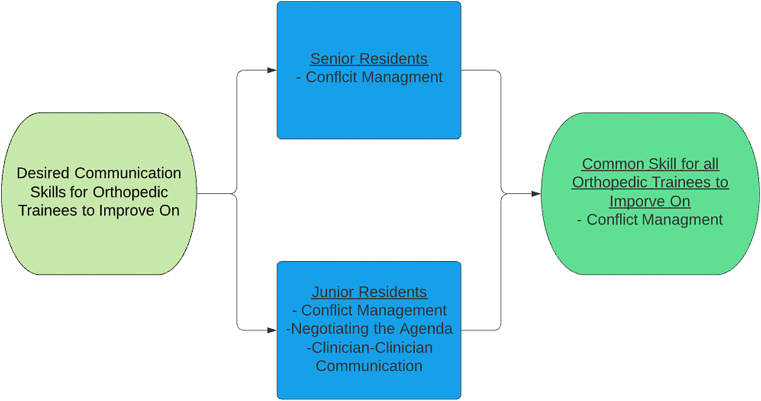
Empirical flowchart of desired skills to improve on by PGY (color). (For interpretation of the references to colour in this figure legend, the reader is referred to the Web version of this article.)

When asked about specific communication skills (Fig. 2), residents wanted to improve on conflict management (71%) and negotiating the agenda (58%). The majority of interns wanted to improve conflict management (80%), negotiating the agenda (80%), creating rapport (80%), and clinician-clinician communication (80%). For comparison, the majority of PGY2-4s sought improvement in all the same areas except creating rapport. The only communication skill that the majority of PGY5s wanted to improve on was conflict management (80%) (Fig. 3).

When asked about communication practices (Table 1), the majority of all residents most often did not do the following: negotiate with patients to establish the agenda (71%), consistently elicit patients’ concerns (79%), offer opportunities to express emotions (67%), and encourage patients to summarize what was discussed (88%).

Regarding communication barriers in the orthopedic surgery service, only one resident agrees/strongly agrees that morning rounds are calm (4%), while 79% agree/strongly agree that they often feel rushed on morning rounds and that orthopedics is one of the busiest services (71%). In terms of specific communication barriers, 12.5% of residents agreed/strongly agreed that there is sufficient time to address patient concerns and 25% agreed/strongly agreed that the quantity of work on rounds promotes communication. Most residents do not agree/strongly disagree that the EMR optimizes communication (75%) or that non-English speaking patients are easily accommodated (88%). Despite the above communication barriers, 83% of residents believe that they treat patients with courtesy and respect.

## 4 Discussion

Our detailed demographic assessment demonstrated that our study sample was diverse in race, gender, and PGY. Because of this, the results of our study may be generalizable to other orthopedic surgery programs. This study highlights the compelling need for a structured communication skills curriculum for orthopedics residents given its current absence from curricula [5]. Results of our needs assessment indicate orthopedics residents want formal communication training.

Junior residents desired training in many more areas than senior residents (e.g., negotiating the agenda and creating rapport) indicating a need for communication training early in residency. Perhaps, PGY5s pick up on these skills informally through direct observation of their predecessors [5]. Interestingly, a study of surgical residents found senior residents were more confident in their communication skills compared to junior residents, but evaluation of these skills did not show better skills amongst senior residents [18].

Despite differences between junior and senior residents’ needs, all residents wanted training in conflict management. A similar need for conflict management training was identified for medicine residents [19] and for ophthalmology residents [20]. While study respondents favored an online method of course delivery, potentially due to time constraints, formal communication training through simulation has proven successful amongst ophthalmology and surgery residents [19,20].

A limitation of this study is that it was performed at a single institution in a single surgical sub-specialty residency program and may not be generalizable to other institutions or surgical subspecialties. Barriers to implementing communication curriculum include time constraints/volume of work and other compelling needs (consults, responding to traumas, etc.). Additionally, not all communication challenges such as working within an EMR and with non-English speaking patients can be addressed with curricular intervention given complex systemic barriers.

## 5 Conclusion

The purpose of this study was to perform an inpatient communication needs assessment on orthopedic surgery residents at our institution. This assessment will establish the foundation of any future RCC curriculum. Our results showed a need among the orthopedics residents for an inpatient communication skills training curriculum early in residency, specifically in conflict management. We also found significant differences in the communication goals between junior and senior residents. Early curricular intervention utilizing the principles of RCC may address these communication needs.

## Ethical approval

This study was approved by Stanford University Institutional Review Board as an exempt protocol.

## Sources of funding

This study was funded by the Department of Orthopaedic Surgery, 10.13039/100005492Stanford University, CA, United States. The funding was used to pay for a gift card for survey respondents.

## Author contributions

Aussama K. Nassar: study concept or design, data collection, supervisor, discussion, writing the paper, editing, submitting.

Drew Thomas: study concept or design, data collection, discussion, writing the paper, editing.

Raffi Avedian: study concept or design, data collection.

Tyler Johnson: study concept or design, discussion, writing.

Marisa Kline: study concept or design, writing.

Barbette Weimer-Elder: study concept or design, writing.

John B Michaud: writing, discussion.

## Trial registry number

Name of the registry: researchregsitry.com.

Unique Identifying number or registration ID: researchregistry7370.

Hyperlink to your specific registration (must be publicly accessible and will be checked):

https://researchregistry.knack.com/research-registry#user-researchregistry/

## Guarantor

Aussama K. Nassar.

## Consent

Not a case review.

## Provenance and peer review

Not commissioned, externally peer-reviewed.

## Declaration of competing interest

No conflict of interests for the entire team.
