# Supplementary material for: Education research - Understanding the factors involved in inpatient communication for orthopedic trainees
Source: Ann Med Surg (Lond). 2021 Nov 19;72:103079. doi: 10.1016/j.amsu.2021.103079 (PMC8632834; doi:10.1016/j.amsu.2021.103079)
Supplement: Multimedia component 1 [file mmc1.docx]

[Supplemental]

Table: Barriers to Communication: Percent that agree/ strongly agree

| Item | PGY2  (*n*=8) | PGY3  (*n*=4) | PGY4  (*n*=2) | PGY5  (*n*=5) | Intern  (*n*=5) | Total *(n=24)* |
| --- | --- | --- | --- | --- | --- | --- |
| *Quantity of work on rounds promotes communication*    *Morning rounds are calm/easeful*    *I often feel rushed on morning rounds*    *There is sufficient time to address patients’ concerns*    *There are multiple interruptions most days*    *There is adequate time to obtain a treatment plan*    *EMR documentation allows for optimal communication*    *Having bedside nurses on rounds would be beneficial*    *Non-English-speaking patients are easily accommodated*    *Orthopedics is one of the busiest services*    *My main goal on rounds is to gather objective medical data*    *We treat our orthopedics patients with courtesy and respect*    *Orthopedics does a great job of listening to patients*    *We give explanations understandable to patients*    *We explain new medications and side effects every time*    *We explain new tests/imaging every time* | 50    12    87    25    37    75    50    25    37    75    62    100    50    100    25    25 | 0    0    50    0    25    0    0    0    0    75    25    50    0    50    0    25 | 50    0    100    0    0    0    50    100    0    50    50    50    50    0    0    50 | 20    0    100    0    12    60    20    20    0    80    60    80    20    80    0    60 | 0    0    60    20    80    20    0    80    0    60    100    100    80    0    20    20 | 25    4    79    12.5    36    42    25    37.5    12    71    62    83    42    58    12.5    33 |

Provenance and peer review
Not commissioned, externally peer-reviewed
